# Follicular Fluid from Cows That Express Estrus During a Fixed-Time Artificial Insemination Protocol Promotes Blastocyst Development

**DOI:** 10.3390/jdb13020014

**Published:** 2025-04-25

**Authors:** Audra W. Harl, Verónica M. Negrón-Pérez, Jacob W. Stewart, George A. Perry, Alan D. Ealy, Michelle L. Rhoads

**Affiliations:** 1 School of Animal Sciences, Virginia Tech, Blacksburg, VA 24061, USA; aharl@shastacollege.edu (A.W.H.); veronica.negron4@upr.edu (V.M.N.-P.); jstewart23@radford.edu (J.W.S.); ealy@vt.edu (A.D.E.); 2 Texas A&M AgriLife Research and Extension Center, Overton, TX 75684, USA; george.perry@ag.tamu.edu

**Keywords:** cattle, embryo, follicular fluid, estrus, fertility, pregnancy

## Abstract

It is not yet understood why cows that exhibit estrus and ovulate are more likely to become pregnant than those that ovulate but do not exhibit estrus during a fixed-time artificial insemination (FTAI) protocol. The objective of this work was to determine whether the follicular fluid from cows that exhibit estrus contributes to the increased likelihood of pregnancy. Lactating crossbred cows were subjected to an FTAI estrous synchronization protocol. Estrous behavior was observed and recorded prior to transvaginal follicle aspiration from cows that did (estrus, *n* = 7) or did not exhibit estrus (non-estrus, *n* = 6). Follicular fluid (25%) was then added to in vitro maturation media for the maturation of oocytes (*n* = 1489) from slaughterhouse ovaries. Cleavage rates were not affected by the estrous status of the cows from which the follicular fluid was collected. Blastocyst rates, however, were greater following maturation in the presence of follicular fluid from estrus cows compared to non-estrus cows (*p* ≤ 0.01). This difference in blastocyst rates was not related to blastocyst cell numbers (inner cell mass, trophoblast, and total), as they did not differ between estrus and non-estrus animals. This study demonstrates that the follicular fluid, and thus, the follicular environment just prior to ovulation does indeed contribute to improved pregnancy rates following FTAI.

## 1. Introduction

For any cattle breeding operation, profitability depends on the success of reproductive management programs. The development and implementation of fixed-time artificial insemination (FTAI) protocols have largely eliminated the need for estrous detection, reducing labor costs while increasing the number of animals inseminated via AI [1]. While FTAI protocols increase breeding efficiency, estrous detection remains one of the most straightforward indicators of correct response to a synchronization protocol. The purpose of an FTAI protocol, however, is to eliminate the need for estrous detection by inducing ovulation of the dominant follicle with an injection of gonadotropin-releasing hormone (GnRH), regardless of estrus expression [2].

Even though ovulation is induced during an FTAI program, there is an opportunity for cows to exhibit estrus, prior to the final injection of GnRH. It has been well documented that animals who display estrous behavior have greater fertility when compared with animals not displaying estrus [3,4,5,6]. Logically, one’s first inclination is to assume that a higher proportion of cows displaying estrus have properly responded to the injections administered during the FTAI protocol, which would explain the improvement in fertility. The removal of non-responders from the calculations does not negate the effect, however, indicating that there is something about the display of estrus that provides a reproductive advantage. Meta-analysis of five FTAI protocols reported that animals displaying estrus experienced a 27% increase in conception compared with animals that did not show estrus [1]. Circulating estradiol concentrations and follicle size were also greater in animals that displayed estrus during FTAI protocols [7]. Even two months after AI, those cows that had displayed estrus prior to FTAI continued to outperform their counterparts as they were less likely to experience pregnancy loss when compared with animals not displaying estrus [4]. Up to this point, only limited research investigating the mechanisms responsible for the observed improvement in fertility has been conducted. None, however, have examined the role of the follicular fluid from cows that did or did not express estrus on the maturing cumulus–oocyte complex.

In vivo, the follicle-enclosed oocyte matures and prepares for ovulation surrounded by follicular fluid. Follicular fluid plays a crucial role in nuclear and cytoplasmic maturation of the oocyte as well as final follicle maturation and ovulation. Because of its contact with the oocyte and surrounding granulosa cells, follicular fluid components can serve as indicators and/or drivers of oocyte quality and competence [8,9]. As the follicle and oocyte grow and develop, the composition of the follicular fluid undergoes dynamic changes [10] and ultimately differs between those animals that display estrus and those that do not [1,11,12,13]. Differences in the characteristics of the follicular fluid could be affecting the ability of the oocyte to successfully complete maturation and later develop into a competent embryo, thereby causing the observed differences in pregnancy rates based on the display of estrus. Therefore, the purpose of this study was to evaluate whether oocyte maturation and subsequent embryo development are affected by follicular fluid, specifically follicular fluid from cows that display estrus compared to those that do not display estrus during FTAI.

## 2. Materials and Methods

This study was conducted at the Virginia Tech beef cattle facilities at Kentland Farm with the approval of the Institutional Animal Care and Use Committee. Thirty multiparous, lactating Angus crossbred cows were housed on mixed grass pastures with ad libitum access to grazing and water. Body condition score (BCS) and body weight (BW) were collected at the beginning of the experiment.

### 2.1. Estrous Synchronization and Detection

**Figure 1:**
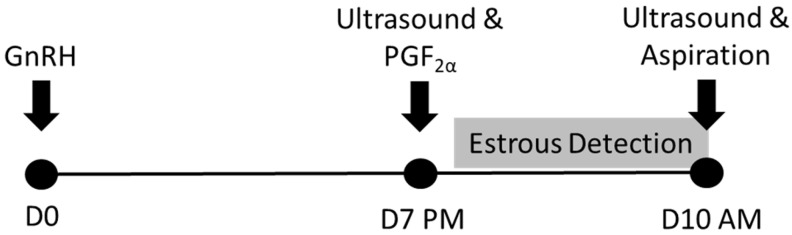
Schedule of injections for estrous synchronization, transrectal ultrasonography, estrous detection, and dominant follicle aspiration. Behavioral estrous detection began 12 h after the PGF_2α_ injection and was conducted every 12 h until dominant follicle aspiration.

The timeline of treatments administered to cows is depicted in Figure 1. Cows were subjected to the CoSynch estrous synchronization protocol. Briefly, cattle were administered GnRH (100 µg of Cystorelin, Merial Ltd., Iselin, NJ, USA) on d 0. On d 7, the ovaries of each animal were examined via transrectal ultrasonography (Ibex ^®^Pro, E.I Medical Imaging, Loveland, CO, USA) using a 7.5 MHz linear rectal transducer to verify the presence of a corpus luteum. Prostaglandin (PG) F_2α_ was then administered (25 mg of Lutalyse, Pfizer Animal Health, New York, NY, USA), and tail paint (DetectHer, H&W Products, Salem, OH, USA) was applied liberally to the tailhead of each animal.

Animals were observed for estrous behavior beginning 12 h after the administration of PGF_2α_. Estrous detection consisted of watching the animals for at least 30 min every morning and every evening as well as visually observing the amount of paint remaining on the tailhead. Animals displaying behavioral signs of standing estrus with ≤25% of their tail paint remaining prior to follicle aspiration were categorized as having expressed estrus.

### 2.2. Follicular Fluid Collection and Steroid Measurements

Irrespective of estrous status, the dominant ovarian follicle was measured via transrectal ultrasonography and aspirated approximately 60 h following PGF_2α_ administration. Contents of the dominant follicle were collected via transvaginal aspiration as previously described [14] using a 17-gauge aspiration needle (Cook Medical, LLC, Bloomington, IN, USA) and a 5.0 MHz convex-array transducer (ALOKA 500, Aloka Co., Ltd., Wallingford, CT, USA). The collected samples were centrifuged for the removal of cellular contents. The decanted fluid was then placed on ice for transport to the laboratory where it was frozen and stored at −20 °C until used in in vitro procedures.

The concentration of progesterone in follicular fluid was measured in a single assay using a commercially available radioimmunoassay kit (Coat-A-Count, Siemens Medical Solutions Diagnostics, Los Angeles, CA, USA) [15] with an intra-assay CV of 3.7%. Estradiol concentrations within the follicular fluid were also measured in a single assay by validated radioimmunoassay [16] with an intra-assay CV of 4.74%.

### 2.3. Media Preparation

Maturation media were prepared as experimental treatments. Positive control oocyte maturation medium (OMM; C+) was the medium that our laboratory normally uses for in vitro maturation. It was included in the experiment (and run in each replicate) to ensure that the in vitro system was working properly and so that the quality of each replicate could be assessed. The C+ OMM consisted of TCM-199+ Earls Salts (Gibco 11150-059; ThermoFisher Scientific, Waltham, MA, USA), 10% FBS (ThermoFisher 10437010; ThermoFisher Scientific), 1.14% Glutamax 100× (Gibco 35050-061: ThermoFisher Scientific), 1.14% sodium pyruvate (Gibco 11360-070; ThermoFisher Scientific), 40 µg/mL follicle-stimulating hormone (Folltropin^®^, AgTech, Inc.; Manhattan, KS, USA), estradiol (E2758-1G; Sigma-Aldrich Inc., St. Louis, MO, USA), 50 µg/mL gentamycin (Sigma-Aldrich Inc.), and EGF (E9644-.5MG; Sigma-Aldrich Inc.) and was not supplemented with follicular fluid. Negative control (C− medium contained no undefined ingredients and consisted of TCM-199+ Earls Salts (Gibco 11150-059; ThermoFisher Scientific), 1.14% Glutamax 100× (Gibco 35050-061;ThermoFisher Scientific), 0.3% PVP (PVP40-50G; Sigma-Aldrich Inc.), and 1.14% sodium pyruvate (Gibco 11360-070; ThermoFisher Scientific). This C− OMM served as the base medium for all experimental treatments, which consisted of 75% C− OMM supplemented with 25% follicular fluid from individual cows.

### 2.4. Oocyte Collection, Maturation, and In Vitro Embryo Production

Bovine ovaries were collected from an abattoir (Brown Packing, Gaffney, SC, USA) and transported to the laboratory in 0.9% saline supplemented with penicillin (100 units/mL) and streptomycin (100 units/mL). Follicular content was collected from antral follicles (2–7 mm diameter) of abattoir-derived ovaries via slashing into approximately 150 mL of oocyte collection medium (OCM). The medium was filtered through a 0.2 µm cell strainer to concentrate cumulus–oocyte complexes (COCs); then, the filter material was rinsed onto a gridded plate and examined under a dissecting microscope in order to visualize and collect COCs. The oocytes with healthy, multiple layers of cumulus cells were collected and washed twice in fresh OCM and randomly assigned to treatment maturation media (formulation described above). Oocytes were matured in groups of 15 in 50 µL OMM drops overlaid with mineral oil (Origio, Målov, Denmark) for 21 h at 38.5 °C under 5% CO_2_.

In vitro maturation, fertilization, and embryo culture were performed in 11 replicate experiments. There were not enough oocytes available for each cow’s follicular fluid to be included in each replicate, so cows were alternated from replicate to replicate in a manner that prevented the same cows from being tested side by side exclusively. Furthermore, follicular fluid from individual animals was assigned to replicates so that both estrus and non-estrus animals were included in each replicate. The total COCs for each treatment were 161 C+, 130 C− 626 estrus, and 572 non-estrus.

After being subjected to treatments during the maturation period, all COCs were treated the same for the remainder of the procedures. In vitro fertilization (IVF) and in vitro culture (IVC) media were both prepared as previously described [17,18]. After 21 h maturation, COCs from the same treatment were pooled, washed three times in HEPES-Tyrode’s albumin lactate pyruvate [HEPES-TALP; HEPES-TL (Caisson Laboratories, Inc.; North Logan, UT, USA) supplemented with 3 mg/mL BSA (Fraction V), 22 µg/mL sodium pyruvate, and 75 µg/mL gentamicin], and fertilized in plates containing 500 mL of IVF-TALP [IVF-TL (Caisson Laboratories, Inc.) supplemented with 6 mg/mL BSA (essentially fatty acid free), 22 µg/mL sodium pyruvate, 10 µg/mL heparin, and 50 µg/mL gentamicin]. Frozen-thawed semen straws from two B. taurus bulls were pooled, purified with BoviPure-BoviDilute 40% [*v*/*v* and 80% (*v*/*v*)], and diluted to a final concentration in the fertilization dishes of 1 × 10^6^/^mL^. Fertilization time was 18–22 h in a humidified gas atmosphere of 5% (*v*/*v*) CO_2_ and 19% (*v*/*v*) O_2_ at 38.5 °C for all groups.

Putative zygotes were collected, exposed to hyaluronidase (Sigma-Aldrich Inc., 1000 U/mL in ~0.5 mL HEPES-TALP), and vortexed for 5 min to remove cumulus cells. They were then washed three times in HEPES-TALP and placed in groups of 15 zygotes per 25 µL drop of synthetic oviductal fluid—bovine embryo 2 (SOF-BE2) covered with mineral oil in a humidified gas atmosphere of 5% (*v*/*v*) CO_2_, 5% (*v*/*v*) O_2_, and the balance nitrogen at 38.5 °C. Cleavage rates were assessed on day 3 and blastocyst rates on day 8 post-fertilization. A replicate was defined as the COC collected in one day for in vitro fertilization procedures. Only replicates with a cleavage rate of ≥70% in the C+ group were included in the data analysis.

### 2.5. Immunofluorescence

After 8 days of culture, blastocysts were collected for cell counting. Only one control group was included in this analysis (C−) because all previous analyses indicated similar performance of the C+ and C− groups. Immunolabeling protocols were conducted as previously described [18]. All immunolabeling procedures were performed at room temperature unless otherwise noted. Briefly, for labeling with CDX2, embryos were placed in permeabilization solution [Dulbecco’s phosphate-buffered saline (DPBS) + polyvinylpyrrolidone (PVP) containing 0.25% (*v*/*v*) Triton X-100] and incubated for 30 min and were then transferred to blocking solution (5% *w*/*v*) bovine serum albumin (BSA) in DPBS for 1 h. Embryos were then transferred to mouse monoclonal anti-human antibody against CDX2 (0.4 µg/mL; CDX2-88, Biogenex; Fremont, CA, USA) and incubated for 1 h in the dark. Embryos were then washed three times in washing buffer [DPBS + 0.1% BSA (*w*/*v*) and 0.1% (*v*/*v*) Tween-20] and transferred to the secondary antibody, conjugated goat polyclonal anti-mouse IgG (1 μg/mL fluorescein isothiocyanate, Abcam, Cambridge, MA, USA), for an incubation period of 1 h in the dark. Embryos were washed three times and counterstained with 1 μg/mL Hoechst 33342 in DPBS-PVP for 15 min, after which they were washed once in DPBS-PVP. Embryos were then transferred to a 10 μL drop of SlowFade Gold antifade reagent (Life Technologies S36936; ThermoFisher Scientific) on a glass microscope slide and covered with a coverslip. To determine non-specific labeling, primary antibodies were replaced with rabbit or mouse IgG (1 μg/mL).

Images of embryos were captured with a 40× objective using a Nikon Eclipse Ti fluorescence microscope (Melville, NY, USA). ImageJ v 1.51n (National Institutes of Health, Bethesda, MD, USA) was used to measure images and count the number of cells.

### 2.6. Statistical Analyses

Development data were analyzed for the main effect of treatment using SAS statistical software version 9.4 (SAS Institute Inc.; Cary, NC, USA). The treatment effect was assessed using PROC GLIMMIX, with the primary dependent variables including cleavage of embryos, development to blastocyst, inner cell mass cell number, trophoblast cell number, and total cell number. The day 3 cleavage rate was calculated as the number of cleaved embryos divided by the total number of COCs subjected to each treatment and the d 8 blastocyst rate was calculated in two manners: the number of blastocysts divided by the total number of COCs subjected to each treatment and the number of blastocysts divided by the number of cleaved zygotes. The random effect of the replicate was included in the model statement. Days postpartum, BW, BCS, follicle diameters, and steroid concentrations were also compared between estrus and non-estrus cows using the GLIMMIX procedure of SAS. Separation of means was conducted with the LSMEANS statement in SAS with the Tukey adjustment. The results are reported as least squares means ± standard error of the mean. Statistical significance was declared at *p* ≤ 0.05.

## 3. Results

Cows were closely observed for behavioral signs of estrus between the PGF_2α_ injection and the time of follicle aspiration. This period coincides with the time during which cows that are being prepared for FTAI may express estrus. Based upon behavioral observations and changes in tail paint, 50% of the 30 cows originally synchronized exhibited estrus. Of the thirty cows that were subjected to the experimental protocol, follicular fluid from the dominant follicle was successfully collected from seventeen of them, and thirteen of those were used in the in vitro experiments (*n* = 7 estrus, *n* = 6 non-estrus), while four were deemed unusable due to visible blood contamination.

Cows categorized as estrus or non-estrus did not differ in BW, BCS, or days postpartum (Table 1; *p* > 0.10). Follicle diameter was determined on d 7 of the protocol, just prior to PGF_2α_ administration. The diameter of the largest follicle tended to be greater in animals that subsequently displayed estrus compared to those that did not (Table 1; *p* ≤ 0.10). Ovarian structures were again measured just prior to aspiration of the dominant preovulatory follicle, and the diameter of the dominant follicle was greater in those animals that displayed estrus than in those that did not (Table 1; *p* ≤ 0.01). The change in follicle diameter between the first and second ultrasound was found to not differ between those cows that did or did not exhibit estrus (Table 1; *p* > 0.10). Despite the difference in follicle size on the day of aspiration, follicular fluid estradiol concentrations did not differ between estrus and non-estrus animals (Table 1; *p* > 0.10). Follicular fluid progesterone concentrations tended to be greater in the cows that displayed estrus than in cows that did not (Table 1; *p* ≤ 0.10).

**Figure 2:**
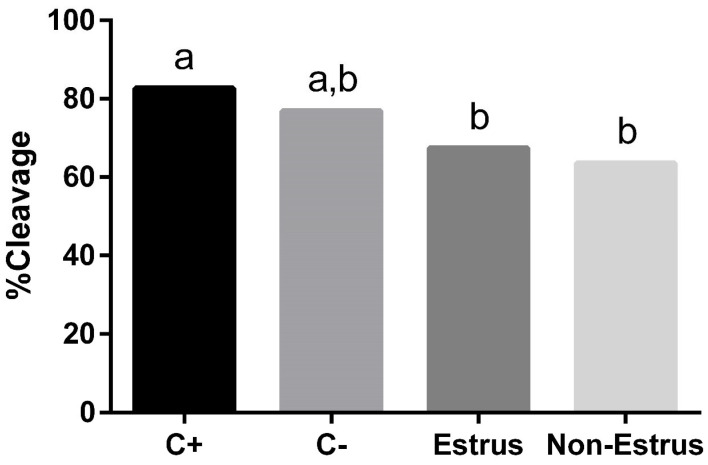
Cleavage rates of embryos resulting from cumulus–oocyte complexes that had been matured in media containing follicular fluid from cows that did (estrus) or did not (non-estrus) exhibit estrus prior to dominant follicle aspiration. Two control groups were included, designated as positive control (C+; normal maturation medium) or negative control (C–; maturation medium with no undefined ingredients). ^a,b^ *p* ≤ 0.01.

Overall, embryo cleavage was affected by treatment (*p* ≤ 0.01), but pairwise comparisons revealed no difference in embryo cleavage between animals displaying estrus when compared with animals not displaying estrus (Figure 2). Instead, differences were driven by the C+ treatment. Oocytes matured in the C+ treatment exhibited greater cleavage rates than either treatment containing follicular fluid (estrus and non-estrus groups; *p* ≤ 0.01). Cleavage within the C– treatment did not differ from any of the treatments.

**Figure 3:**
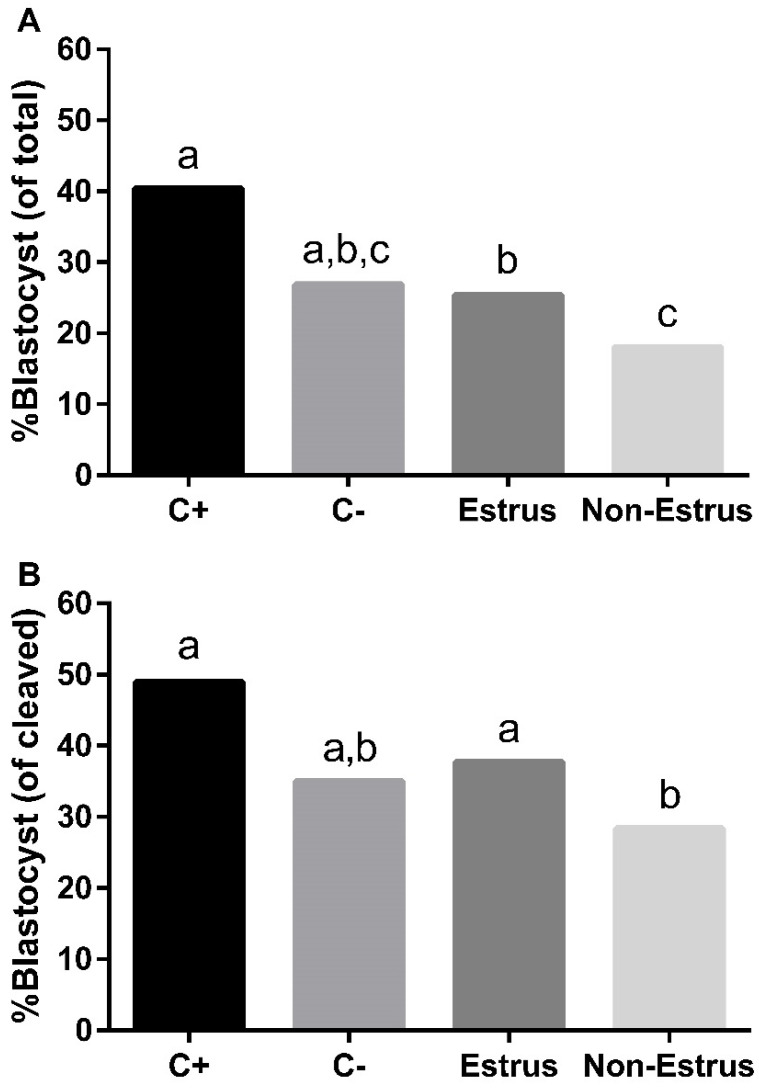
Blastocyst rates resulting from cumulus–oocyte complexes (COCs) that had been matured in media containing follicular fluid from cows that did (estrus) or did not (non-estrus) exhibit estrus prior to dominant follicle aspiration. Two control groups were included, designated as positive control (C+; normal maturation medium) or negative control (C–; maturation medium with no undefined ingredients). Blastocyst rates were calculated (**A**) as a percentage of total COCs subjected to each treatment and (**B**) as a percentage of cleaved COCs from each treatment. ^a,b,c^ *p* ≤ 0.05.

Blastocyst rates were calculated as both a percentage of total COCs and as a percentage of COCs that were cleaved. Regardless of the method of calculation, blastocyst rates were affected by treatments (*p* ≤ 0.01 and *p* ≤ 0.01 for percent of total and percent of cleaved, respectively; Figure 3). The C+ treatment yielded the greatest blastocyst rates (Figure 3), which did not differ from the C– treatment in either analysis. Importantly, the blastocyst rates were consistently greater in the estrus treatment group than the non-estrus treatment group (*p* ≤ 0.05 and *p* ≤ 0.05 for percent of total and percent of cleaved, respectively; Figure 3).

Overall, the number of cells found within the blastocyst inner cell mass on day 8 post-fertilization tended to differ between treatments (Table 2; *p* ≤ 0.10), but similar to cleavage rates, pairwise comparisons revealed no differences between estrus and non-estrus groups. The C− treatment tended to be greater than the non-estrus group (*p* ≤ 0.10) but did not differ from the estrus treatment (Table 2). Trophoblast and total cell counts did not differ amongst any of the treatments (Table 2).

## 4. Discussion

Fixed-time artificial insemination is an important management tool used frequently by cattle producers. It offers many advantages over other reproductive programs, not the least of which is the ability to get cows pregnant without having to check for estrus. Many previous studies have demonstrated, however, that even after excluding cows that fail to ovulate, behavioral estrus is important for maximizing pregnancy rates from FTAI [3,4,5,6]. The mechanisms responsible for these observations have not been defined but likely involve interactions between prevailing hormone profiles, reproductive tissues, and the COCs or early embryos. The results of the current study demonstrate the importance of the preovulatory follicular environment associated with estrus (specifically, the follicular fluid) for maximal fertility in an FTAI program.

Follicle diameter was measured at two different time points in the current study. At the time of PGF_2α_ injection, the cows that exhibited estrus tended to have larger follicles than those that did not exhibit estrus. Despite similar growth rates of the follicles between the PGF_2α_ injection and the second measurement, the dominant follicles of the estrus animals were ultimately larger than those of the non-estrus animals at the time of aspiration. The size of the follicle is directly related to the number of theca and granulosa cells that are responsible for producing the hormone that causes estrous behavior, estradiol. It is not surprising, then, that the animals with larger ovarian follicles were more likely to display estrus. Follicle size has indeed been positively correlated with estradiol concentrations [19,20,21]. This finding highlights the importance of follicle size for fertility during estrous synchronization programs.

The measurement of hormone concentrations within the follicular fluid was limited to estradiol and progesterone in the current experiment in order to spare as much fluid as possible for inclusion in in vitro maturation media. As previously mentioned, follicular fluid estradiol concentrations are important because of their role in inducing estrus and have been measured in experiments examining the importance of estrus for fertility [3,7]. No differences in follicular estradiol concentrations were observed in this experiment, but this is not unexpected since the follicular fluid was collected 60 h after the PGF_2α_ injection. By this time, cows would have been well past peak estradiol concentrations [7]. When measured in serial blood samples collected at regular intervals, previous research has reported greater peak estradiol concentrations in cows that exhibit estrus compared to those that do not [7]. Elevated peak estradiol concentrations have been associated with improved fertilization, embryo quality, and pregnancy establishment [19,22,23]. Estradiol positively influences oocyte developmental and metabolic competency as well as biological regulation of reproductive tissues, thereby affecting synchrony in the oviduct and uterine receptivity to the embryo [24,25,26,27]. Preovulatory estradiol concentrations are undeniably important for subsequent fertility in FTAI programs as they confer both direct (oocyte) and indirect (oviduct and uterus) effects on reproductive processes.

In the present study, progesterone concentrations within the follicular fluid were greater in cows that exhibited estrus than those that did not. Given the timing of the sample collection, these results are consistent with expected changes in the preovulatory follicle. The surge in luteinizing hormone (LH) occurs shortly after the initiation of standing estrus [28,29]. The LH surge initiates the cascade of events that will lead to ovulation approximately 24 h later [29] and also stimulates luteinization of the theca and granulosa cells, causing an increase in progesterone production before the follicle even ruptures [30,31,32]. About 6 h after the LH surge, estradiol concentrations decrease, followed by an increase in progesterone. By 18 h post-LH surge, the follicular steroid concentration is composed of about 90% progesterone [30]. In the present study, most of the cows that exhibited estrus would have experienced the LH surge long before dominant follicle aspiration, resulting in a shift of intrafollicular steroid concentrations from predominantly estradiol to progesterone.

In addition to estradiol and progesterone, there is a myriad of components found in follicular fluid, any of which could be important for the estrus-induced improvement in fertility during FTAI. Follicular fluid is similar to blood plasma and is enriched with substrates locally produced by the theca interna and granulosa cells. In the follicle, sodium, chloride, and potassium concentrations are on a gradient, suggesting an inward transport from serum to the follicle [8]. Glucose, a major energy source for ovarian cells, varies depending on stage of growth and increases as follicular diameter increases [33]. Protein in follicular fluid is about 75% of that of serum and does not appear to differ based on follicle size or stage of the estrous cycle, suggesting that a substantial portion of the protein content of follicular fluid is derived from serum [34,35].

Triglycerides are present in relatively high concentrations in small follicles and decrease as the follicle grows. Levels of triglycerides in small follicles are higher than in blood serum, suggesting that this component is differentially regulated in some manner. Furthermore, triglyceride concentrations in the follicular fluid do not change with diet or physiological status as they do in serum [8,36]. In follicular fluid, triglycerides may serve as an alternate energy source as oocytes and embryos cultured with triglycerides will absorb and metabolize triglycerides from the culture medium [37].

Cholesterol in follicular fluid is present at approximately 42% of serum concentration, and as follicle size increases, cholesterol concentrations increase. Cholesterol present in follicular fluid is bound to high-density lipoprotein, and the increased concentration of cholesterol in large follicles is thought to accumulate due to the increased permeability of the follicle wall, allowing larger molecules to pass through the follicle wall [36].

These components are just a few examples of the factors that could be affecting the COC leading up to ovulation. As the follicle and oocyte grow and develop, the composition of the follicular fluid undergoes dynamic changes [10], and the timely progression of these changes leading up to ovulation is undoubtedly important for the ultimate competency of the COC. Follicular fluid plays a crucial role in nuclear and cytoplasmic maturation of the oocyte as well as final follicle maturation and ovulation [38,39]. Because of its intimate contact with the oocyte and surrounding granulosa cells, follicular fluid components can serve as an index for the function of the follicle and the COC [8,9].

In the current study, the effects of the composition of the follicular fluid were tested in vitro using naïve COCs that were collected from follicles that had not been affected by estrus or an LH surge. This approach was advantageous as it isolated the effects of the follicular fluid from any inherent differences in the COC of estrus vs. non-estrus animals. It also allowed us to examine the effects of the follicular fluid on a much larger number of COCs than if we had used the COC aspirated from the synchronized cattle. The maturation of COCs in the follicular fluid of estrus and non-estrus cows yielded no difference in cleavage rates but did affect blastocyst rates. Regardless of the method of calculation, blastocyst rates were greater for COCs that were matured in follicular fluid from estrus cows compared to non-estrus cows. These results provide definitive evidence that the composition of the follicular fluid just prior to ovulation in an FTAI program affects the competency of the COC and subsequent pregnancy rates. It is worthwhile noting that this experimental design is limited by the fact that the composition of the follicular fluid was static and not reflective of in vivo dynamic changes that could affect the quality of the COC. Until the components of follicular fluid have been fully defined, however, recreating those dynamic changes in vitro will be all but impossible.

## 5. Conclusions

The results of this study provide evidence that the follicular fluid of cows that display estrus prior to ovulation in an FTAI program positively affects the COC in a manner that ultimately improves subsequent development to the blastocyst stage. The specific component(s) responsible for the increase in blastocyst development remains unknown, but taken together, these results highlight the importance of generating a relatively large, highly functional dominant follicle during an estrous synchronization program in order to maximize pregnancy rates. Future research aimed at defining the components of the follicular fluid, their dynamic nature, their interactions, and their impact on oocyte competence will improve our understanding of the importance of follicular fluid for fertility.

## Figures and Tables

**Table 1:** Characteristics of cows that did (estrus) or did not (non-estrus) exhibit estrus prior to dominant follicle aspiration.

	Estrus	Non-Estrus	*p*-Value
Days Postpartum	73.7 ± 3.3	65.2 ± 4.0	0.12
Body Weight (kg)	601.0 ± 27.0	599.7 ± 33.0	0.98
Body Condition Score	5.8 ± 0.2	5.3 ± 0.3	0.19
Initial Follicle Diameter (mm) ^a^	8.32 ± 1.41	4.71 ± 1.35	0.08
Final Follicle Diameter (mm) ^b^	17.55 ± 0.79	13.29 ± 0.76	≤0.01
Change in Follicle Diameter (mm)	9.23 ± 1.29	8.58 ± 1.23	0.72
Follicular Fluid Estradiol (ng/mL)	355.00 ± 144.22	381.63 ± 166.53	0.91
Follicular Fluid Progesterone (ng/mL)	153.14 ± 30.73	66.21 ± 33.19	0.08

^a^ Ovarian follicle diameter at the time of prostaglandin F_2α_ injection. ^b^ Ovarian follicle diameter at the time of dominant follicle aspiration.

**Table 2:** Cell counts of embryos (day 8 post-fertilization) resulting from cumulus–oocyte complexes that had been matured in media containing follicular fluid from cows that did (estrus) or did not (non-estrus) exhibit estrus prior to dominant follicle aspiration.

	C– ^a^	Estrus	Non-Estrus	*p*-Value
Inner Cell Mass	42.7 ± 8.2	27.5 ± 1.6	24.9 ± 2.0	0.09
Trophoblast	109.3 ± 17.5	84.8 ± 3.3	83.4 ± 4.4	0.36
Total Cell Number	152.0 ± 22.8	112.3 ± 4.4	108.3 ± 5.7	0.18

^a^ C− = negative control group.

## Data Availability

The data presented in this study are available upon request from the corresponding author.
